# Psoriasis: An Immunogenetic Perspective

**DOI:** 10.1055/s-0042-1743259

**Published:** 2022-06-13

**Authors:** Ayca Kocaaga, Mustafa Kocaaga

**Affiliations:** 1 Department of Medical Genetics, Eskişehir City Hospital, Eskisehir, Turkey; 2 Department of Medical Microbiology, Yunus Emre State Hospital, Eskisehir, Turkey

**Keywords:** autoimmune, genome-wide association study, immunogenetics, psoriasis

## Abstract

Psoriasis is an erythematous-squamous dermatosis with a polygenic inheritance history. Both environmental and genetic factors play a role in the etiology of the disease. Over the past two decades, numerous linkage analyzes and genome-wide association studies have been conducted to investigate the role of genetic variation in disease pathogenesis and progression. To date, >70 psoriasis susceptibility loci have been identified, including HLA-Cw6, IL12B, IL23R, and LCE3B/3C. Some genetic markers are used in clinical diagnosis, prognosis, treatment, and personalized new drug development that can further explain the pathogenesis of psoriasis. This review summarizes the immunological mechanisms involved in the etiopathogenesis of psoriasis and recent advances in susceptibility genes and highlights new potential targets for therapeutic intervention.

## Introduction

**Fig. 1:**
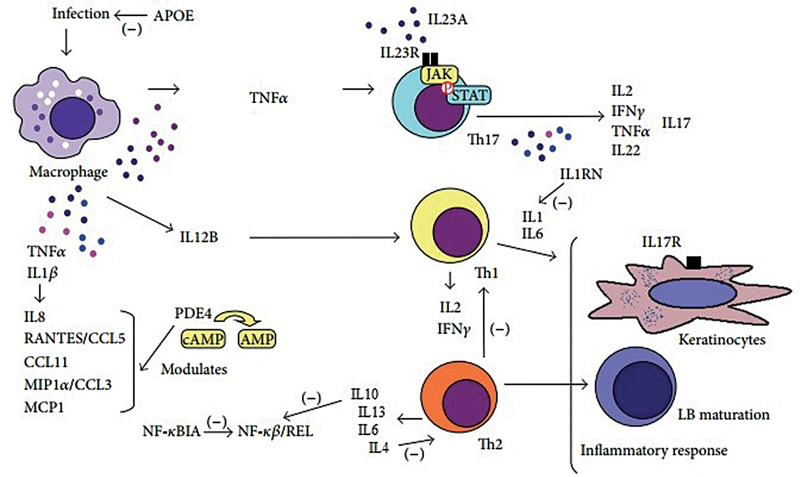
Summary of cytokine involvement in the pathogenesis of psoriasis.

Psoriasis is a clinically common chronic inflammatory disease characterized by skin tissue damage and concomitant other systemic complications. 1 2 Although psoriasis is more common in American, Canadian, and European populations, it is seen all over the world affecting ∼1 to 3% of the world's population. 3 This disease usually presents with clinical and histological features such as adherent, raised silver scales, dividing lines, and oval-shaped plaques with erythema. 4 Psoriasis is considered to occur through chronic interactions between hyperproliferative keratinocytes and activated immune cells. In recent years, cellular and molecular contributions have been demonstrated in response to an overactive immune response. 5 Since psoriasis is a skin-specific autoimmune disease, cytokines, chemokines, adhesion factors, epidermal growth factors, nerve growth factors, and especially Th1 and Th17 polarization play a role in its pathogenesis ( Fig. 1 ). 6 Although the exact cause of psoriasis is unknown, its genetics are complex and multifactorial. In this article, we summarize what is currently known about the immunogenetics of psoriasis pathogenesis.

## The Immunogenetics of Psoriasis

### HLA-C

The first gene known to be susceptible to psoriasis is HLA-Cw6, located at chromosome location 6p21 (PSORS1). HLA-C encodes an major histocompatibility complex (MHC) class I receptor involved in the immune system and is involved in the presentation of antigens to CD8+ T lymphocytes. 7 In recent years, many studies have been conducted to examine the contribution of the HLA-Cw6 allele to the pathogenesis of psoriasis. 8 The prevalence of HLA-Cw6 varies worldwide, ± 16% in Africa, 8.5 to 12% in Europe, and 3.5 to 7.8% in Asia. 9 In a study, psoriasis was also associated with HLA-C*12:03. 10 The HLA-C*12:02 allele was related with late-onset disease in Japanese patients. 11

### ERAP1

The endoplasmic reticulum aminopeptidase 1 ( *ERAP1* ) gene, which belongs to the M1-aminopeptidase family, is located on chromosome 5q15. ERAP1 is involved in processing peptides for MHC class I presentation. 12 A genome-wide association study (GWAS) demonstrated a significant interaction between the HLA-Cw6 allele and the rs27524 ERAP1 polymorphism. 13 ERAP1 is involved in processing peptides for MHC class I presentation. The rs30187 (C/T) and rs27524 (G/A) polymorphisms of ERAP1 were found to be associated with increased risk of psoriasis in a Chinese cohort. 14 A recent meta-analysis showed rs27524 and rs30187 polymorphisms and susceptibility to psoriasis, while lack of association was obtained for rs26653 and rs27044 polymorphisms. 15

### LCE and CDSN

The PSORS4 locus, located on chromosome 1q21, contains genes that continue without completing epidermal differentiation complex (EDC) formation and keratinization. The late keratinized envelope (LCE) gene cluster is located in the PSORS4 locus of chromosome 1q21.3 and is a part of the EDC. 16 The copy number variation (LCE3C_LCE3B-del) in the LCE cluster was linked with psoriasis in British, Italian, and Spanish populations, but it was not repeated in German or Tunisian cohorts. 17 18 19 20 Interaction between the MHC and LCE was found in Chinese and Dutch populations, the combination of risk alleles in both of the MHC and LCE genes was showed to increased psoriasis. 21 22 The *CDSN* gene encodes corneodesmosin, and in the process of keratocyte maturation, the encoded proteins undergo a succession of cleavages and are localized to human epidermis. The CDSN allele 5 (+619T, +1240G, +1243C) was linked to susceptibility with psoriasis in Caucasian but not in Japanese populations. 23 A meta-analysis showed no significant association between CDSN -619C/T polymorphism and susceptibility to psoriasis in Caucasian and Asian patients. 24 The minor allele (A) of (PSORS1C1/CDSN) rs1062470 was shown to increase the disease risk of psoriasis. 25

### KLF4, DEFB4, and GJB2

Kruppel-like factor 4 (KLF4) is a transcription factor involved in a variety of cellular events, including development, differentiation, proliferation, and apoptosis. In a functional study, KLF4 expression was shown significantly reduced in psoriatic compared with healthy cells. 26 A GWAS demonstrated that KLF4 was a likely gene for susceptibility of psoriasis. 27 An increased copy number of DEFB4 has been associated with psoriasis in a study with Dutch psoriasis patients. 28 DEFB4 gene transcription has been shown to be strikingly reduced in psoriatic keratinocytes of psoriasis patients. 29 *GJB2* gene encodes connexin 26, a gap junction protein expressed at high levels in psoriatic keratinocytes. 30 GJB2 showed evidence for association with the German replication cohort, but it was not replicated in an American cohort. 31 A recent study showed that the CT (rs3751385, GJB2) genotype was protective in late-onset male psoriasis vulgaris, as well as the T allele in female early-onset psoriasis vulgaris. 32

### IL-1 Gene Family

Moorchung et al found that there was a strong association between the interleukin (IL)-1β C/C genotype and psoriasis. 33 A of study by Tarlow et al showed that the frequency of the A2 allele of IL1RN VNTR increased in the early-onset (<40 years) cohort with psoriasis. 34 A meta-analysis result emphasized that there is no relationship between IL1RN VNTR and psoriasis pathogenesis. 35 A GWAS showed that the (rs397211) IL1RN polymorphism was associated with psoriasis susceptibility. 36

### IL-2, IL-4, IL-6, IL-10, and IL-13

A study showed that rs2069762 (G allele) in IL-2 conferred a risk of developing the disease, mainly in late-onset psoriasis in a Japanese cohort. 37 The rs20541 of IL-4 was found to be associated with psoriasis in a Caucasian population. The GG genotype of IL-6 -174G > C polymorphism was found to be associated with twofold increased risk of the psoriasis. 36 Xu et al revealed that two single-nucleotide polymorphisms (SNPs) in IL-6R (rs4845617 and rs2228145) demonstrated an association with psoriasis. 38 The presence of C allele in the IL-6 SNP rs1800795 decreased the risk of psoriasis. 39 Moreover, a recent meta-analysis showed that the IL-6 -174G/C polymorphism contributes to psoriasis risk. 40 Ahmed et al found a higher serum level of IL-6 in Egytptian patients with psoriasis compared to controls. 41 Craven et al found a significant difference in rs1800896 (IL-10) genotype frequencies between patients and controls. 42 A meta-analysis study indicated that -1082 G/A(rs1800896) polymorphism confers susceptibility to psoriasis in the Asian population, but there was no risk in Europeans. 43 There was an association found between rs20541 in IL-13 and psoriasis. 44 The CCG haplotype of rs1800925, rs20541, and rs848 of IL-13 was found to be associated with susceptibility to psoriasis. 45

### IL-15, IL-17A, IL-17F, and IL17RA

The polymorphisms in IL-15 (rs2857261, rs10519613, and rs1057972) have been associated with psoriasis in a Chinese subject. 46 Sanad et al showed that the frequency of GA + AA genotypes of IL-17A was significantly higher in psoriasis cases than in controls. 47 The T allele and TT genotype of the IL-17F rs763780 polymorphism were associated with a decreased risk of psoriasis. 48 The IL-17F His161Arg polymorphism was significantly associated with psoriasis based on the genotype and allele analyses in an Asian population. 49 The IL17RA promoter region (rs4819554) was associated with psoriasis susceptibility in Egyptian psoriatic patients. 50 A study with Spanish population demonstrated the SNP rs4819554 in the promoter region of IL17RA significantly influenced the response to anti-tumor necrosis factor (TNF) drugs. 51

### IL-18, IL-19, IL-20, and IL20RA

The minor alleles of the IL-19 gene SNPs (rs2243188, rs2243169, and rs2243158) revealed protective effect to psoriasis and the TGATA haplotype in *IL-19* gene proved significant protective effect. 52 The G allele of rs1713239 (IL-20) was associated with psoriasis susceptibility in a Chinese population. 53 Kingo et al revealed an association between rs2981572 (IL-20) and predisposition to psoriasis in Caucasian patients. 54 Moreover, the haplotype in IL-19 and IL-20 was associated a risk factor for the development of psoriasis. 55 The IL-20 T allele (rs1400986) was found to be linked to protection from psoriasis. 56 In addition, the polymorphisms in the IL-20 receptor (IL20RA) have also been associated with psoriasis. 57

### IL-12, IL-22, IL-23, and IL23R

A GWAS showed a reported psoriasis-associated SNP in the IL12B 3′ untranslated region (rs3212227). This study also identified two missense SNPs (rs7530511 and rs11209026) in IL23R that associated with psoriasis. 58 Capon et al found a significant difference between the psoriasis patients and control groups for rs3212227 in IL12B. 59 A GWAS with a Caucasian population revealed an association between SNPs in IL23R (rs7530511 and rs11209026) and IL12B (rs6887695 and rs3212227) and predisposition to psoriasis. 60 Liu et al found an association between the IL23R (rs11209026) and IL12B (rs6887695) polymorphisms and psoriasis. 61 Another recent study also showed a link between rs11209026 of *IL23R* gene and psoriasis. 44 A GWAS in Caucasian patients showed the rs2201841 and rs2066808 (IL23R) and rs2082412 and rs2546890 (IL12B) polymorphisms were associated with psoriasis. 36 The A allele (rs3212227, IL12B) was found more frequent in Japanese patients with psoriasis than in healthy controls. 62 A GWAS with Chinese population found that the rs6887695 IL12B SNP was associated with psoriasis. 63 The rs7530511 and rs3212227 ( *IL23R* gene) polymorphisms were associated with psoriasis in a Thai cohort. 64 The nonsynonymous SNP in IL23R, rs11209026, widely thought to be the primary psoriasis-associated SNP in IL23R in Europeans, was found not to be polymorphic in Chinese. 65 66

### TNF-α and TGF-β1

The TNF-α polymorphisms (rs1800629 and rs361525) linked a strong association in Caucasian patients with early-onset psoriasis. 67 A meta-analysis study showed that when the GA + AA genotype was compared with the GG genotype, the risk of psoriasis increased for rs361525 and decreased for rs1800629 in *TNF-α* gene. 68 Moreover, a functional study found an association between the A allele in rs361525 in the *TNF-α* gene and increased production of TNF-α and early-onset psoriasis. 69 A study with an Egyptian case–control revealed an association between TNF-α (GG allele in rs1800629) polymorphism and psoriasis. 41 Another study with Caucasian patients showed decreased frequency of the GG genotype and increased frequency of the GA genotype of rs361525 ( *TNF-α* gene) in patients with type I (onset before 40 years) psoriasis compared with controls. 70 A recent meta-analysis showed that TNF-α -238 G/A, -308 G/A, and -857 C/T polymorphisms were associated with elevated susceptibility to psoriasis in certain populations. 71 *TGF-β1* gene polymorphism at codon 10 (T869C) is significantly associated with susceptibility to psoriasis in Egyptian patients. 72 *TGF-β1* gene polymorphism in codons 10 and 25 are not associated with susceptibility to psoriasis vulgaris in Polish patients. 73

### TNFAIP3 and TRAF3IP2

TNFAIP3 interacting protein (TNIP1) regulates the activity of nuclear factor kappa B (NF-κB). The rs610604 (TNFAIP3) and rs17728338 (TNIP1) SNPs were associated with psoriasis in a case–control study. 44 Ellinghaus et al found an association between two SNPs (rs13210247 and rs33980500) in NF receptor-associated factor 3 interacting protein gene and psoriasis. 74 Hüffmeier et al replicated the association in a German population with psoriasis. 75 A significant association between psoriasis and the SNP rs610604 of *TNFAIP3* gene was found in an Egyptian cohort. 76 In a GWAS, the rs240993 (TRAF3IP2) was associated with psoriasis in Caucasian patients. 13 A meta-analysis demonstrated that rs610604 in TNFAIP3 and rs17728338 in *TNIP1* gene polymorphisms were associated with psoriasis susceptibility. 77

## Toll-Like Receptors

A study with a Turkish population demonstrated that the TLR2-rs4696480 AA genotype seemed to have a higher risk for psoriasis. 78 Zabłotna et al found no statistically significant association between Arg753Gln TLR2 and -1237 T/C *TLR9* gene polymorphisms and psoriasis in a Polish cohort. 79 The SNP rs3804099 of TLR2 was linked to psoriasis susceptibility in a Chinese population. 80 A study from Turkey demonstrated that GA genotype and A allele in TLR2 Arg753Gln polymorphism were associated with psoriasis. 81

### APOE, ACE, ANGPT2, VDR, MTHFR, and VEGF

Apolipoprotein E (APOE) alleles ε2, ε4, and genotypes ε2/ε3 and ε4/ε3 were found to be a risk factor for psoriasis, while allele ε3 and genotype ε3/ε3 were associated to be protective factor for psoriasis in a Saudi cohort. 82 A meta-analysis demonstrated that the ε2 and ε3 alleles of the APOE polymorphism were associated with the risk of psoriasis. 83 A meta-analysis showed that the homozygous I/I genotype and I allele increased risk of psoriasis, while the heterozygous I/D genotype decreased risk in Asian but not in Caucasian populations. 84 The results of the another meta-analysis showed that angiotensin-converting enzyme I/D polymorphism may be associated with psoriasis susceptibility, while ID genotype seemed to have a protective role in Caucasian patients affected by psoriatic arthritis. 85 The rs2442598 polymorphism of angiopoietin-2 was significantly associated with psoriasis. 86 The polymorphisms of vitamin D receptor has been found the conflicting results in psoriasis. A meta-analysis showed that the vitamin D receptor (VDR) *Taq* I polymorphism was associated with psoriasis susceptibility in Caucasian populations. This meta-analysis also indicated the polymorphisms in VDR ApaI, BsmI, and FokI were not associated with psoriasis susceptibility in Caucasian or Asian populations. 87 Huraib et al showed that the T allele and TT, CT genotypes of methylenetetrahydrofolate reductase (MTHFR) C677T are significantly linked with psoriasis susceptibility. 88 A meta-analysis showed that there is no association between MTHFR C677T polymorphism and either Asian or European psoriatic patients. 89 The -1154 G allele and +405 CC and -460 TT genotypes of vascular endothelial growth factor ( *VEGF* ) gene demonstrated that there is a significantly increased risk of psoriasis in a Polish cohort. 90 A meta-analysis demonstrated the VEGF +405 C/G polymorphism susceptibility to psoriasis in Asians, and the -460 C/T and -1154 A/G polymorphisms susceptibility to psoriasis in Europeans. 91

## Inflammasome-Related Genes

The polymorphism rs10403848 in CARD8 was significantly associated with psoriasis risk in the Chinese Han population. 92 A CARD11 rs4722404 SNP was also associated with increased risk of early-onset psoriasis in a Chinese population. 93 Moreover, the CARD10 SNPs were not association with psoriasis in another Chinese population. 94 The rs11652075 CC (p.Arg820Trp) genotype of CARD14 was significantly associated with psoriasis in a Spanish cohort. 95 The CARD14 c.526G > C (p.Asp176His) polymorphism was found to be a significant risk factor for generalized pustular psoriasis in a Japanese cohort. 96 In a study with a Chinese patients, the CC genotype of c.C2458T SNP in the CARD14 was related and associated significantly with an increased familial history with psoriasis. 97 GWASs have found the SNP c.C2458T in *CARD14* gene was associated with psoriasis. 98 The association between NOD2/CARD15 polymorphisms and psoriasis was not found in a meta-analysis. 99 The NLRP1 rs8079034C and rs878329C alleles were associated to be a risk factor for psoriasis. 100 NLRP3 rs10733113 and CARD8 rs2043211 polymorphisms were associated with psoriasis in a Swedish population. 101 Two SNPs, rs3806265 and rs10754557, in NLRP3 were significantly associated with psoriasis in a Chinese Han population. 102

## The Other Genes

Kim et al showed JAK2 rs7849191polymorphism was a protective factor for psoriasis in the Korean population. 103 Sayed et al found a possible association between *JAK1* rs310241 and *JAK3* rs3008 gene polymorphisms and susceptibility to psoriasis. 104 The genotypes of rs744166GG in STAT3 and rs7574865TT in STAT4 were found higher in psoriasis patients than the controls in Northeastern China. 105 The rs1020760 at NF-κB1 was associated with family history of psoriasis in a Chinese cohort. 106 The rs2847297, rs657555, and rs482160 polymorphisms of *PTPN2* gene were significantly associated with psoriasis. 107 The (1858C/T) R620W polymorphism of PTPN22 was found to be positively linked with susceptibility of psoriasis in Saudis. 108 A study with a Turkish population demonstrated that Vaspin rs2236242 polymorphism was related to psoriasis. 109 A meta-analysis demonstrated that the CD143 ID polymorphism linked the risk of psoriasis in individuals with East Asian. 110 The G allele of PD1.6 increased the risk of psoriasis in the Chinese subjects. 111 The frequency of the rs2787094 C allele of *ADAM33* gene was significantly linked with psoriasis in a Han population. 112 The *HCP5* gene 335 T> G polymorphism was associated with the increased risk of developing psoriasis in the Indian patients. 113

## Conclusion

Psoriasis is an incurable disease that negatively affects the quality of life of affected individuals. Although the exact cause of psoriasis remains unknown, immune factors play a very important role in its pathogenesis. In recent years, more than 70 psoriasis susceptibility loci have been associated. However, the identified genes account for approximately one-third of the heritability of psoriasis, suggesting the existence of additional yet unidentified sources of inheritance. Indeed, in the case of complex hereditary diseases such as psoriasis, it turned out that the identification of genetic risk factors is not sufficient to predict the development of the disease or assess its severity. Current management of psoriatic patients is a real challenge for clinicians. Recent therapeutic recommendations regarding the place of biotherapies are based directly on immunological mechanisms that have recently been elucidated. Advances in biology have certainly made it possible in recent years to elucidate many aspects of the pathogenesis of psoriasis, but have not answered the main questions regarding the nature of the antigen and/or the genes responsible. Answering these questions could lead to the development of more targeted and effective treatments, curative and even preventative treatments.
